# Supplementary figures and images for: Multidimensional Gene Space as an Approach for Analyzing the Organization of Genomes
Source: Int J Mol Sci. 2025 Dec 10;26(24):11926. doi: 10.3390/ijms262411926 (PMC12732336; doi:10.3390/ijms262411926)

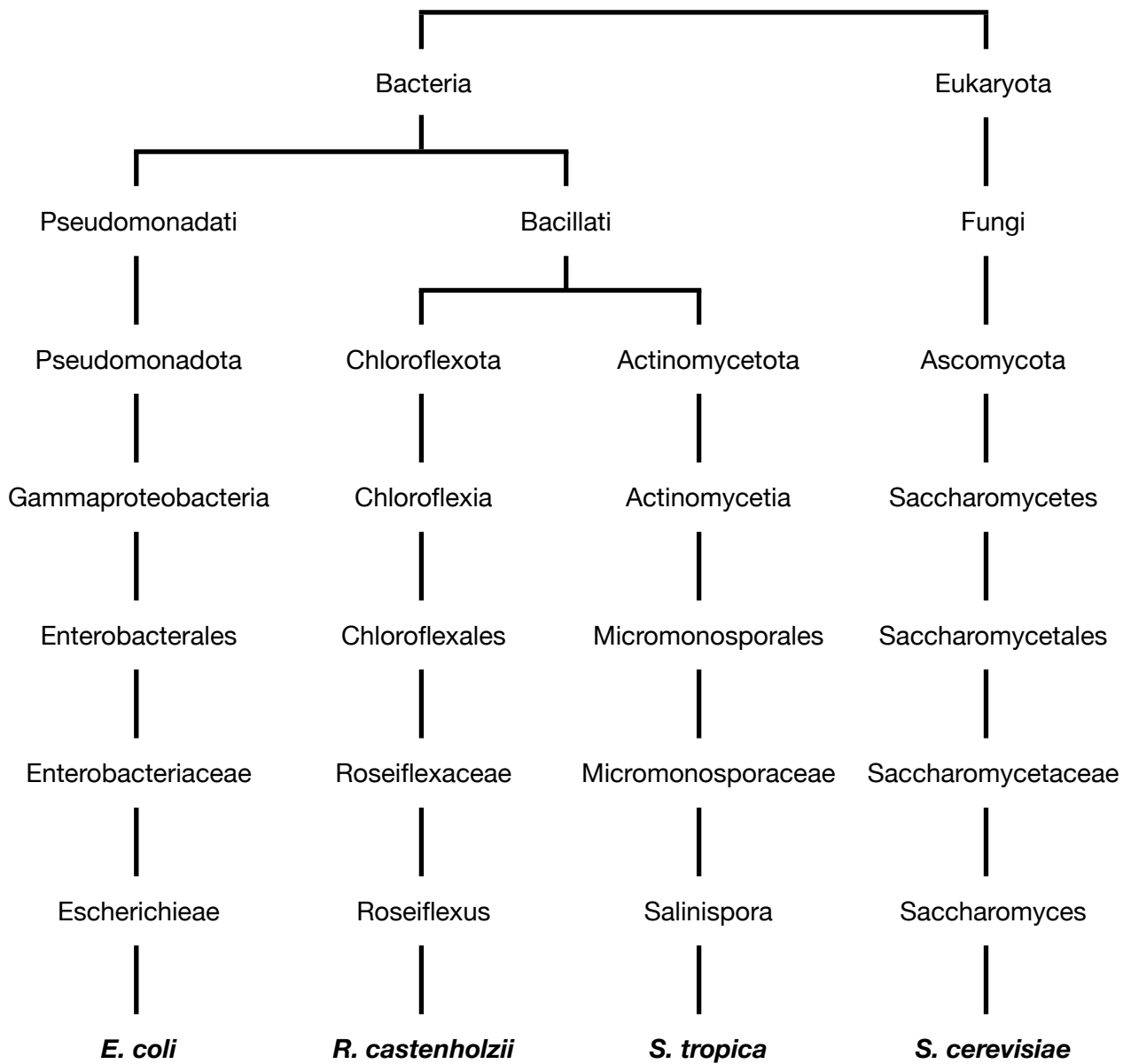

Supplement: Supplementary file 1 [file ijms-26-11926-s001.zip › ijms-3982926-supplementary.pdf]
